# Effect of Oral and Vaginal Hormonal Contraceptives on Inflammatory Blood Biomarkers

**DOI:** 10.1155/2015/379501

**Published:** 2015-03-16

**Authors:** Afshin A. Divani, Xianghua Luo, Yvonne H. Datta, James D. Flaherty, Angela Panoskaltsis-Mortari

**Affiliations:** ^1^Department of Neurology, University of Minnesota, Minneapolis, MN 55455, USA; ^2^Division of Biostatistics, School of Public Health, University of Minnesota, Minneapolis, MN 55455, USA; ^3^Division of Hematology, Oncology, and Transplantation, Department of Medicine, University of Minnesota, Minneapolis, MN 55455, USA; ^4^Division of Cardiology, Department of Medicine, Feinberg School of Medicine, Northwestern University, Chicago, IL 60611, USA; ^5^Blood and Marrow Transplantation Division, Department of Pediatrics, University of Minnesota, Minneapolis, MN 55455, USA

## Abstract

The use of combined hormonal contraceptives has been reported to increase the level of C-reactive protein (CRP). We assessed the effect of hormonal contraceptive use on inflammatory cytokines including CRP, monocyte chemotactic protein-1, soluble tumor necrosis factor (sTNF), interleukin-6 (IL-6), and soluble CD40 ligand. We used 79 female subjects (19 to 30 years old) who were combined oral contraceptives users (*n* = 29), combined vaginal contraceptive users (*n* = 20), and nonusers (*n* = 30) with CRP values of ≤1 (*n* = 46) or ≥3 (*n* = 33). Information on medical history, physical activities, and dietary and sleeping habits were collected. Both oral and vaginal contraceptive users had higher levels of CRP (*P* < 0.0001), compared to nonusers. Only oral contraceptive users exhibited elevated sCD40L (*P* < 0.01). When comparing the groups with CRP ≤ 1 and CRP ≥ 3, levels of IL-6 and sTNF-RI were positively correlated with CRP among oral contraceptive users. We did not observe the same elevation for other inflammatory biomarkers for the CRP ≥ 3 group among vaginal contraceptive users. The clear cause of elevation in CRP level due to the use of different hormonal contraceptive formulations and methods is not well understood. Longitudinal studies with larger sample size are required to better assess the true cause of CRP elevation among hormonal contraceptive users.

## 1. Introduction

Inflammatory processes play an underlying role in pathogenesis of vascular events. With atherosclerosis recognized as such a process [1, 2], several plasma markers of inflammation have been evaluated as potential tools for prediction of the risk of vascular events. Among them are markers of systemic inflammation produced in the liver such as C-reactive protein (CRP), serum amyloid A, cytokines such as interleukin-6 (IL-6), monocyte chemotactic protein-1 (MCP-1), and adhesion molecules such as soluble intercellular adhesion molecule type 1 (sICAM-1) [3–6]. The circulating level of CRP is commonly used as an inflammatory marker to assess the risk for cardiovascular disease (CVD) and stroke [7–13]. The significance of elevated CRP as a marker of inflammation in the clinical setting has been suggested in the literature [6, 14, 15]. Published studies suggest a correlation between proinflammatory cytokines related to severity of the atherosclerotic process and CRP levels [13].

The use of combined oral contraceptives (COCs) elevates the level of circulating CRP [16–20]. However, the definite underlying cause is not clear. One recent hormonal contraceptive method is combined vaginal contraceptive (CVC), commercially marketed as NuvaRing (Merck & Co., Inc., Whitehouse Station, NJ). The use of CVCs bypasses the absorption of the steroids through the gastrointestinal tract, avoiding immediate hepatic first-pass metabolic effects [21, 22]. Our objective for conducting this study was to assess changes in inflammatory markers due to use of COC and CVC that may lead to vascular disease.

## 2. Methods

The Institutional Review Board at the University of Minnesota approved the study. Detailed study information on design and subject recruitment has been described previously [23, 24]. Briefly, women between 19 and 30 years of age were recruited in three groups: (1) nonuser subjects: women who had not been on any hormonal contraceptive for a minimum of six months, (2) COC users: women who have been using pills for a minimum of six months, and (3) CVC users: women who have been using vaginal rings (NuvaRing) for a minimum of six months. Detailed description of different COCs used by the subjects in our study has been given previously [24]. The exclusion criteria consisted of subjects with a recent history of viral or bacterial infection, history of recent surgery, history of chronic inflammatory diseases, history of malignancy, history of pregnancy within six months prior to the study, and history of antiplatelet/anticoagulant medications within one week prior to the study.

With consent provided, the subjects were asked to fill out a standardized questionnaire describing their medical history, familial medical history, physical activities, dietary and sleeping habits, and general lifestyle choices. A brief exam was also conducted to record blood pressure (BP), pulse rate, body temperature, weight, height, and waist and hip circumferences to calculate waist to hip ratio. Body mass index (BMI) was defined as weight/height^2^.

Following the subject examination, phlebotomy was performed to collect whole blood in sodium citrate, ethylenediaminetetraacetic acid (EDTA), and serum separator Vacutainer tubes. The citrated and EDTA whole blood samples were centrifuged at 1000 g for 15 minutes to collect plasma samples. Serum separator tubes were allowed to clot for 30 minutes and then centrifuged at 1000 g for 15 minutes. All samples were appropriately aliquoted and labeled for storage at −80°C for batch analysis.

Tests for complete blood count (CBC) with differentials (i.e., neutrophils, lymphocytes, monocytes, eosinophils, and basophils), D-dimer, fibrinogen concentration, and ultrasensitive C-reactive protein (CRP, cardiac risk) concentration were completed by the University of Minnesota-Fairview Acute Care Clinical Laboratory [24]. Plasma and serum samples were tested using the following enzyme-linked immunosorbent assays (ELISA): chemokine (C-C motif) ligand 2 (CCL2), also referred to as MCP-1, with 5 pg/mL sensitivity (R&D Systems, Minneapolis, MN), IL-6 with 0.039 pg/mL sensitivity (R&D Systems, Minneapolis, MN), and soluble CD40 ligand (sCD40L: BD, Franklin Lakes, NJ). Levels of soluble tumor necrosis factor (sTNF) receptor type I (sTNF-RI, sensitivity 5.0 pg/mL) and receptor type II (sTNF-RII, sensitivity 25.0 pg/mL) were determined by multiplex analysis using human specific bead sets from Millipore (Billerica, MA) on the Luminex platform (Austin, TX) with Bioplex software (BioRad, Hercules, CA).

### 2.1. Statistical Analysis

All continuous measurements were analyzed for departure from normality. For normally distributed variables, mean and standard deviation (mean ± SD) were reported and *F*-tests and *t*-tests were used for 3- and 2-group comparisons, respectively. Median and interquartile ranges were reported for nonnormally distributed variables and Kruskal-Wallis and Wilcoxon rank sum tests were used for comparisons. For categorical variables, Chi-square or Fisher's exact tests were used as appropriate. In addition to study groups, all multivariate regression models were adjusted for covariates, including age, race, alcohol consumption, regular sleeping habits, and family history of cardiovascular disease (CVD) and stroke. We have reported the least square means for different groups and the *P* values for different contrasts, based on the estimated regression models. The association between CRP and other inflammatory biomarkers was investigated by using both the dichotomous CRP levels (≥1 versus ≤3) and potentially more powerful analysis with the continuous CRP. Wilcoxon rank sum test and multivariate regression analysis (adjusted for the same covariates as above) were used for the dichotomous CRP levels. Spearman correlation and partial Spearman correlation (adjusted for the same covariates in the regression analysis) were used for continues CRP analysis. The association analysis was conducted for all the selected recruited subjects as a whole and for subgroups of subjects stratified by their contraceptive use status (nonusers, COC users, and CVC users). A *P* < 0.05 was considered statistically significant. We performed the statistical analyses using SAS 9.3 (SAS Institute Inc., Cary, NC).

## 3. Results

Table 1 provides information such as demographics, vital signs and physical measurements, lifestyle, and medical history of the selected subjects used in this study. For the current study, there were 79 subjects (nonusers = 30, COC users = 29, and CVC users = 20), out of 159 subjects recruited for the original study, who had a CRP value of ≤1 (*n* = 46) or ≥3 (*n* = 33). The nonusers were significantly younger than the COC users (*P* = 0.01) and the CVC users (*P* < 0.0001). They also consumed alcohol significantly less as compared to the COC users (*P* < 0.01) and the CVC users (*P* < 0.0001). Moreover, the nonusers had a significantly lower BP and a lower combined family history of CVD/stroke. Multivariate analysis of differential lymphocytes showed a near-significant difference (*P* = 0.0521) only between the nonusers and COC users. For CRP < 1 there were 29 nonusers, 9 COC users, and 8 CVC users; for CRP > 3 there were 1 nonuser, 20 COC users, and 12 CVC users.

As shown in Table 2, the CRP levels for both COC and CVC groups were significantly higher (*P* < 0.0001 and *P* < 0.001 for COC and CVC users versus nonusers, resp.) after adjusting for age, race, alcohol consumption, sleeping habit, and family history of CVD and stroke. The difference in concentration of sCD40L between the COC users as compared to the CVC users and the nonusers groups was also significant in multivariate analysis.

We analyzed the association between different blood inflammatory biomarkers with CRP level as a dichotomous variable (≤1 versus ≥3) or continuous variable (see Table 3). In the univariate analysis, all blood inflammatory biomarkers showed positive correlation with CRP. Among them, significant correlation was observed between sCD40L and CRP (median sCD40L, 2411 and 3769 for the CRP ≤ 1 and ≥3 groups, resp., *P* = 0.01; Spearman correlation = 0.35, *P* < 0.01). We did not observe any significant association between sTNF-RII and dichotomous CRP values, but a significant correlation was observed with the continuous CRP values (Spearman correlation = 0.24, *P* = 0.04).

In the multivariate analysis (see Table 3), after adjusting for covariates, continuous CRP values and sCD40L still showed significant correlation (partial Spearman correlation = 0.30, *P* < 0.01) even though the comparison between the two dichotomous groups of CRP (≤1 versus ≥3) became nonsignificant (*P* = 0.06) (see Table 3). In addition, multivariate analysis of the continuous variables revealed significant correlation between IL-6 and CRP (partial Spearman correlation = 0.25, *P* = 0.03), whose correlation in the univariate analysis was not significant.

We also investigated the association between CRP and other blood inflammatory biomarkers in the subgroups of subjects stratified by their contraceptive use status (nonusers, COC users, and CVC users). Even though constrained by small sample sizes, we observed significant correlation between sTNF-RII and CRP (Spearman correlation = 0.46, *P* = 0.02; partial Spearman correlation = 0.42, *P* = 0.02) and between sCD40L and CRP (Spearman correlation = 0.40, *P* = 0.05; partial Spearman correlation = 0.36, *P* = 0.05) for the nonusers using a continuous CRP, though the analysis using the dichotomous CRP did not reach significance (see Table 3).

For the COC users (see Table 3), significant association between MCP-1 and CRP (median MCP-1, 153 and 203 for the CRP ≤ 1 and ≥3 group, resp., *P* = 0.04) was observed in the analysis with the dichotomous CRP, but not with the continuous CRP or in the multivariate analysis. IL-6 showed significant difference between the CRP ≤ 1 and ≥3 groups (median IL-6, 0.45 versus 0.76, resp., *P* = 0.05) in the univariate analysis and significant correlation with the continuous CRP in the multivariate correlation analysis (partial Spearman correlation = 0.52, *P* < 0.01). As shown in Table 3, sTNF-RI was significantly different between the two CRP groups (median sTNF-RI, 780 and 1087 for the CRP ≤ 1 and ≥3 group, resp., *P* = 0.04) in the univariate analysis and was found significantly correlated with the continuous CRP in the multivariate analysis (partial Spearman correlation = 0.46, *P* = 0.01) in the COC users. Interestingly, we did not observe any significant difference or association for the CVC users, as shown in Table 3.

## 4. Discussion

In this study, we demonstrated that users of both oral and vaginal contraceptives had elevated levels of CRP, but only users of oral contraceptives had elevated levels of sCD40L (suggesting* in vivo* platelet activation among COC users) [24]. Markers for different inflammatory cytokines such as CRP, TNF-*α*, soluble intercellular adhesion molecule-1 (slCAM-1), serum amyloid A, IL-1, and IL-6 are used in clinical settings to identify individuals with higher risk of vascular events [2, 3, 25–27]. However, evaluating levels of sCD40L among hormonal contraceptive users have been performed for the first time in our study. CRP is part of the innate immune system, binding various ligands for elimination, and activating complement [28]. It is synthesized by hepatocytes in response to IL-6, IL-1*β*, and IL-17. However, it is also synthesized by a number of other cells, but to what degree is unknown [29]. Elevated levels of CRP, due to the use of combined hormonal contraceptives, have been reported in the literature and are in accordance with the findings of our study [16–19, 30, 31].

In a prospective study conducted by Ridker et al. [3] among postmenopausal women, four markers of inflammation (i.e., CRP, serum amyloid A, IL-6, and sICAM-1) were shown to be significantly increased among 122 women with history of cardiovascular events as compared to 224 women (matched for age and smoking status) with no history of cardiovascular events. Based on the obtained results, it was concluded that these four markers were significant predictors of future cardiovascular events, particularly hs-CRP. However, little is known about CRP in young, healthy women and the risk for developing cardiovascular disease.

Plasma level of CRP is considered as a marker of hepatic protein response to acute inflammation. In the case of postmenopausal hormone replacement therapy, lack of effect on markers of white blood cell activation, such as sICAM-1, vascular cell adhesion molecule 1, E-selectin, and IL-6, has led to the hypothesis that elevation of CRP due to oral estrogen may be as a result of metabolic response, rather than an inflammatory response [18, 32]. van Rooijen et al. [19] suggested the same hypothesis by showing higher levels of CRP with unaffected levels of IL-6 and TNF-*α* among COC users. Nonetheless, the hypothesis of direct metabolic hepatic activation as an underlying cause of a rise in CRP levels cannot explain elevation of CRP among CVC users due to their avoidance of hepatic circulation. This may be explained by induction by CVC of CRP by nonhepatocytes [31].

Like CRP and serum amyloid P, pentraxin 3 (PTX-3) is from the pentraxins family and an acute phase protein that is involved with clearance of apoptic cells [33, 34]. Recently, Piltonen at el. [30] have shown elevation of plasma level of PTX-3 among COC and transdermal contraceptive users, suggesting that elevation of CRP may not be just a hepatic byproduct. However, they did not find the same elevation of PTX-3 among CVC users. Unlike the outcomes obtained from our study, another study published by Rad et al. [31] suggested a higher increase in levels of CRP among CVC (Nestorone/ethinyl estradiol, a one-year long-acting ring) users as compared to COC users with no effect on other inflammatory biomarkers. One possible explanation for the observed discrepancy could be the difference between the CVC formulations used in the two studies.

In the present study, we selected subjects from our previous study [24] based on an outcome-dependent sampling method [35] to better identify the correlation between elevated levels of CRP and other inflammatory biomarkers used in our study. Subjects were selected from the two extreme ends of CRP values (≤1 and ≥3) to better observe any correlation with the other inflammatory markers. In the selected subjects, we had only one nonuser with CRP ≥ 3. Compared to the nonusers, we observed a significantly elevated level of sCD40L for the COC users, but not for CVC users. CRP plays a key role in humoral- and cell-mediated immunity and is increased in the setting of elevated cytokines such as TNF-*α* or IL-1*β* and is associated with chronic inflammatory diseases. Elevation of both CRP and sCD40L levels is reported in hypertensive subjects [36]. Higher levels of sCD40L concentration among COC users may be due to an observed higher level of lymphocytes, since CD40L is also found on the surface of B cells [24]. In our study, the difference in lymphocytes count was not significant (*P* = 0.0521) between the nonusers and COC users groups. Alternatively, elevated serum level of sCD40L is a marker of platelet activation, which plays an important role in the pathophysiology of acute coronary syndromes [37]. Published studies suggest a significantly increased risk of developing future myocardial infarction and stroke among healthy women and patients with a history of atrial fibrillation who have a high concentration of sCD40L [38, 39].

In our stratified analysis, we did find a correlation between elevated levels of CRP with IL-6 and sTNF-RI in COC users only. Inflammatory cytokine IL-6 is secreted by macrophages and T cells as a response to inflammation and is considered as one of the crucial cytokines regarding cardiovascular disease. Elevated levels of circulating IL-6 promote myocardial injury progression [4]. Both TNF-RI and TNF-RII are receptors for TNF-*α* and are almost ubiquitously expressed. TNF-RI is constitutively expressed and is the dominant, high affinity receptor that can be activated by soluble TNF-*α* as well as membrane-bound TNF-*α* (i.e., nonsecreted). TNF-RII is a lower affinity receptor that is modulated by inflammation that is activated by membrane-bound TNF-*α*. The levels of the soluble TNF-Rs remain relatively stable in healthy subjects (but vary between individuals), and shedding increases in response to TNF-*α* and thus they are sensitive biomarkers of inflammation. Therefore, a more informative use of sTNF-R levels may be realized in comparing levels before and during use of COCs or CVCs within the same individual subjects.

Elevated levels of inflammatory cytokines such as IL-6 and TNF-*α* are associated with increases in MCP-1 (CCL2), which is a major mediator of monocyte chemotaxis to areas of inflammation. Many cell types secrete MCP-1 and, most relevant to this study, these include endothelial cells and vascular smooth muscle cells, especially when stimulated by combinations of IL-1, IL-6, and TNF-*α*. We did not find any correlation of CRP with MCP-1, indicating that either insignificant inflammation was present or CRP is a more sensitive indicator of low level inflammation.

Our study had some caveats. The small sample size population prevented us from obtaining results that can be considered definite, so the presented results should be interpreted cautiously. The nonrandomized nature of type of hormonal contraceptives (pill versus ring) used in our study may have introduced study bias. However, it is interesting to note that, with the dichotomous CRP, there are differences between the groups regarding which additional inflammatory markers are elevated. This may have implications for the predictive value of CRP for cardiovascular risk in young women using different forms of hormonal contraception. The higher levels of CRP may be associated with increased subclinical atherosclerosis that can potentially contribute to future cardiovascular events [40]. Therefore, a thorough investigation of true causes of elevated CRP levels among hormonal contraceptive users is warranted. To do this, longitudinal studies with a sufficient sample size are needed where the blood samples are taken at the same phase of the menstrual cycle to look at short- and long-term effects of contraceptive use on inflammation. These studies are needed to better understand the effect of different contraceptive methods on inflammation in individual users, particularly when combined with other cardiovascular risk factors including smoking and higher age. Better understanding of the sources causing elevated CRP among hormonal contraceptive users can be helpful in the correct interpretation of elevated CRP in other indications.

## 5. Conclusions

The definite cause of elevation in CRP level due to the use of different hormonal contraceptive formulations and methods is not well understood. While CRP levels were elevated among both COC and CVC users in our study, we only observed a significant correlation between elevated levels of sCD40L and IL-6 with CRP in our multivariate analysis, whereas other inflammatory biomarkers did not reveal any association with elevated CRP. Insofar, as CRP is considered a marker of hepatic protein response to acute inflammation, the cause and significance of elevated CRP level among CVC users are unclear, as we did not observe any correlation between elevated CRP levels and other inflammatory biomarkers among CVC users in our study. Longitudinal studies with larger sample size are required to better assess the correlation between elevated CRP values and other inflammatory biomarkers that can also lead to discovering the true pathways that cause CRP elevation for different formulations and methods of hormonal contraceptives. Moreover, in such a study, any significant result can be evaluated vis-à-vis its clinical impact.

## Figures and Tables

**Table 1:** Demographics, vital signs and physical measurements, lifestyle, and medical history of the selected recruited subjects.

Variable	Nonusers (*n* = 30)	COC (*n* = 29)	CVC (*n* = 20)	Global test *P* value	Nonusers versus COC *P* value	Nonusers versus CVC *P* value
Age	20.9 ± 1.8	22.3 ± 2.3	24.2 ± 2.7	<0.0001^*^	0.01^*^	<0.0001^*^
Race				0.04^*^	0.09	0.05
White	20 (67%)	26 (90%)	19 (95%)			
African American	4 (13%)	0	0			
Native American	0	0	1 (5%)			
Asian	4 (13%)	1 (3%)	0			
Other	2 (7%)	2 (7%)	0			
Vital signs and physical measurements						
SBP	111.8 ± 11.3	118.9 ± 11.4	117.0 ± 9.2	0.04^*^	0.02^*^	0.10
DBP	65.9 ± 12.0	72.0 ± 9.8	72.5 ± 9.0	0.04^*^	0.04^*^	0.04^*^
MAP	81.2 ± 10.6	87.6 ± 9.5	87.3 ± 8.5	0.03^*^	0.02^*^	0.04^*^
Pulse rate	79.0 ± 14.5	80.1 ± 11.7	70.9 ± 10.6	0.03^*^	0.74	0.04^*^
BMI	24.1 ± 5.4	22.5 ± 3.7	23.8 ± 3.8	0.40	0.21	0.82
WTH	0.81 ± 0.07	0.83 ± 0.08	0.81 ± 0.07	0.58	0.34	0.91
Lifestyle factors						
Current smoker	0	0	1 (5%)	0.25	1.00	0.40
Alcohol consumption	15 (50%)	26 (90%)	19 (95%)	<0.0001^*^	<0.01^*^	<0.0001^*^
History of marijuana use	2 (7%)	2 (7%)	3 (15%)	0.54	1.00	0.38
Exercise (≥3 hrs/week)	23 (77%)	18 (62%)	15 (75%)	0.42	0.22	0.89
Regular sleeping habit	24 (80%)	27 (93%)	16 (80%)	0.28	0.25	1.00
Regular diet	24 (80%)	22 (76%)	16 (80%)	0.94	0.70	1.00
Medical history						
Heart disease	0	0	1 (5%)	0.25	1.00	0.40
Urinary tract infection	1 (3%)	3 (10%)	4 (20%)	0.16	0.35	0.14
Depression	2 (7%)	6 (21%)	2 (10%)	0.26	0.15	1.00
Thyroid disease	0	1 (3%)	0	0.62	0.49	1.00
Migraine	0	1 (3%)	1 (5%)	0.52	0.49	0.40
History of pregnancy	0	1 (3%)	0	0.62	0.49	1.00
Family history of hypertension	4 (13%)	10 (34%)	10 (50%)	0.02^*^	0.07	<0.01^*^
Family history of CVD or stroke	3 (10%)	9 (31%)	9 (45%)	0.01^*^	0.06	<0.01^*^
Family history of CVD	3 (10%)	9 (31%)	7 (35%)	0.07	0.06	0.07
Family history of stroke	0	3 (10%)	2 (10%)	0.14	0.11	0.16

Note: data are presented as mean ± SD or *n* (%). A *P* value of <0.05 is indicated by ∗.

BMI: body mass index, COC: combined oral contraceptive, CVC: combined vaginal contraceptive, CVD: cardiovascular disease, DBP: diastolic blood pressure, MAP: mean arterial pressure, SBP: systolic blood pressure, and WTH: waist to hip ratio.

**Table 2:** Multivariate analysis of the blood inflammatory biomarkers for the selected recruited subjects.

Blood marker	Least square mean (95% CI)			*P* value			
	Nonusers	COC	CVC	Global test	Nonuser versus COC	Nonuser versus CVC	COC versus CVC
MCP-1 (pg/mL)	285 (195, 375)	189 (104, 274)	161 (53, 270)	0.22	0.14	0.12	0.69
IL-6 (pg/mL)	1.1 (0.8, 1.5)	0.9 (0.6, 1.2)	1.1 (0.6, 1.5)	0.64	0.38	0.83	0.55
sTNF-RI (pg/mL)	975 (845, 1105)	950 (829, 1071)	805 (649, 962)	0.25	0.79	0.13	0.14
sTNF-RII (pg/mL)	4310 (3777, 4842)	4295 (3799, 4791)	4311 (3672, 4951)	1.00	0.97	1.00	0.98
sCD40L (ng/mL)	2937 (1843, 4031)	5259 (4240, 6277)	2806 (1493, 4119)	<0.01^*^	<0.01^*^	0.89	<0.01^*^
CRP (mg/L)	0.6 (−0.2, 1.4)	3.6 (2.9, 4.4)	2.9 (2.0, 3.9)	<0.0001^*^	<0.0001^*^	<0.001^*^	0.24
Lymphocytes (10e^9^/L)	1.93 (1.67, 2.19)	2.31 (2.05, 2.57)	2.27 (1.94, 2.60)	0.14	0.0521	0.15	0.85

Note: a *P* value of <0.05 is indicated by ∗. Multivariate analysis adjusted for age, race, alcohol consumption, regular sleeping habit, and family history of cardiovascular disease (CVD) and stroke.

MCP-1: monocytes chemotactic protein-1, IL-6: interleukin-6, sCD40L: soluble CD40 ligand, sTNF-RI: soluble tissue necrosis factor receptor I, sTNF-RII: soluble tissue necrosis factor receptor II, and CRP: C-reactive protein.

**Table 3:** Univariate and multivariate analysis of the blood inflammatory biomarkers by dichotomized CRP level (≤1 versus ≥3) and correlation and partial correlation between different blood inflammatory biomarkers with continuous CRP level for the selected recruited subjects.

		Univariate analysis					Multivariate analysis				
	Blood marker	Median [interquartile range]			Spearman correlation with continuous CRP		Least square mean (95% CI)			Partial Spearman correlation with continuous CRP	
		CRP ≤ 1	CRP ≥ 3	*P* value	*r*	*P* value	CRP ≤ 1	CRP ≥ 3	*P* value	*r*	*P* value
All Subjects	MCP-1	175 [144–216]	205 [159–296]	0.08	0.16	0.16	228 (161, 296)	206 (122, 290)	0.70	0.07	0.58
	IL-6	0.72 [0.45–1.18]	0.81 [0.48–1.17]	0.46	0.19	0.09	0.98 (0.71, 1.25)	1.09 (0.76, 1.42)	0.62	0.25	0.03^*^
	sTNF-RI	842 [716–995]	875 [725–1238]	0.13	0.17	0.13	865 (770, 961)	1005 (887, 1123)	0.09	0.19	0.11
	sTNF-RII	3989 [3153–5187]	4217 [3670–5172]	0.34	0.24	0.04^*^	4292 (3899, 4685)	4324 (3841, 4807)	0.92	0.20	0.08
	sCD40L	2411 [1693–4456]	3769 [2492–6027]	0.01^*^	0.35	<0.01^*^	3171 (2311, 4031)	4551 (3493, 5608)	0.06	0.30	<0.01^*^

Nonusers^¶^	MCP-1	194 [144–216]	330 [330-330]	0.14	0.39	0.05^*^	230 (96, 364)	724 (−299, 1746)	0.34	0.13	0.51
	IL-6	0.88 [0.46–1.29]	1.82 [1.82-1.82]	0.26	0.26	0.22	1.13 (0.70, 1.56)	3.50 (0.23, 6.77)	0.16	0.31	0.10
	sTNF-RI	927 [769–1091]	1017 [1017-1017]	0.57	0.25	0.23	976 (845, 1106)	917 (−79, 1914)	0.91	0.24	0.21
	sTNF-RII	3978 [3181–4826]	4761 [4761-4761]	0.49	0.46	0.02^*^	4198 (3654, 4741)	5562 (1419, 9704)	0.51	0.42	0.02^*^
	sCD40L	2213 [1680–3109]	3264 [3264-3264]	0.43	0.40	0.05^*^	2934 (1915, 3952)	403 (−7366, 8171)	0.52	0.36	0.05

COC users	MCP-1	153 [111–189]	203 [175–317]	0.04^*^	0.23	0.31	166 (101, 231)	233 (190, 276)	0.11	0.29	0.14
	IL-6	0.45 [0.37–0.78]	0.76 [0.47–1.16]	0.05^*^	0.39	0.07	0.59 (0.09, 1.09)	0.97 (0.65, 1.29)	0.22	0.52	<0.01^*^
	sTNF-RI	780 [667–954]	1087 [822–1324]	0.04^*^	0.25	0.25	869 (625, 1112)	1007 (850, 1163)	0.37	0.46	0.01^*^
	sTNF-RII	4573 [3518–5399]	4180 [3652–4554]	0.91	−0.05	0.81	4339 (3516, 5162)	4236 (3708, 4764)	0.84	0.04	0.86
	sCD40L	4674 [2375–5773]	5799 [3062–7385]	0.34	0.28	0.19	3417 (943, 5891)	6184 (4596, 7772)	0.08	0.16	0.40

CVC users	MCP-1	172 [166–235]	194 [122–230]	0.91	0.02	0.94	189 (132, 246)	190 (144, 236)	0.99	−0.02	0.94
	IL-6	0.72 [0.55–1.38]	0.83 [0.53–1.28]	0.97	−0.25	0.39	1.11 (0.59, 1.62)	0.92 (0.51, 1.33)	0.57	0.14	0.55
	sTNF-RI	616 [534–791]	805 [660–1040]	0.09	−0.03	0.91	696 (476, 916)	854 (678, 1029)	0.27	0.37	0.12
	sTNF-RII	3725 [2531–5640]	4940 [3754–5416]	0.39	0.03	0.91	4529 (3404, 5653)	4394 (3494, 5294)	0.85	0.40	0.09
	sCD40L	2539 [1695–3582]	3101 [1737–3734]	0.73	−0.17	0.57	3528 (1846, 4671)	2642 (1512, 3772)	0.49	0.10	0.69

Note: a *P* value of <0.05 is indicated by ∗. Multivariate and partial Spearman correlation analyses adjusted for age, race, alcohol consumption, regular sleeping habit, and family history of CVD and stroke. ^¶^There was only 1 subject in the nonusers group whose CRP ≥ 3.

## Abbreviations

BMI: Body mass index
BP: Blood pressure
DBP: Diastolic blood pressure
MAP: Mean arterial pressure
SBP: Systolic blood pressure
CBC: Complete blood count
COC: Combined oral contraceptive
CRP: C-reactive protein
CVC: Combined vaginal contraceptive
CVD: Cardiovascular disease
EDTA: Ethylenediaminetetraacetic acid
ELISA: Enzyme-linked immunosorbent assays
IL-6: Interleukin-6
MCP-1: Monocyte chemotactic protein-1
PTX-3: Pentraxin 3
sCD40L: Soluble CD40 ligand
sTNF: Soluble tumor necrosis factors
sICAM-1: Soluble intercellular adhesion molecule type 1
WTH: Waist to hip ratio.
